# Níveis Altos de Tiol: Um Novo Marcador de Arritmias Ventriculares na Síndrome Coronária Aguda?

**DOI:** 10.36660/abc.20210602

**Published:** 2021-09-01

**Affiliations:** 1 Hospital Barra D'Or Rede D'Or São Luiz Rio de Janeiro RJ Brasil Hospital Barra D'Or - Rede D'Or São Luiz, Rio de Janeiro, RJ - Brasil; 2 Universidade Federal do Rio de Janeiro Instituto de Cardiologia Edson Saad Rio de Janeiro RJ Brasil Universidade Federal do Rio de Janeiro - Instituto de Cardiologia Edson Saad, Rio de Janeiro, RJ - Brasil

**Keywords:** Compostos de Sulfidrila, Arritmias Cardíacas, Síndrome Coronariana Aguda

Arritmias ventriculares em pacientes com síndrome coronária aguda não são frequentes, mas levam a um aumento nas ocorrências de complicações e morte.^1^^,^^2^ Sua prevalência é mais alta em infarto com supradesnivelamento do segmento ST (STEMI) e tem um prognóstico pior quando ocorre 48 horas após a admissão.^3^ Portanto, um marcador capaz de prever a ocorrência desses eventos é bem-vindo.

Três mecanismos justificam a ocorrência de arritmias na fase aguda do infarto:

- O miocárdio lesionado, que pode ser um substrato para o desenvolvimento de circuitos de reentrada ou um foco de aumento de automatismo;
- Desencadeamento de arritmias resultantes de variação da frequência cardíaca;
- Fatores moduladores, tais como distúrbios eletrolíticos, disfunção autonômica, isquemia persistente, ou redução da função do ventrículo esquerdo.

Portanto, a identificação dos marcadores que influenciam esses mecanismos pode ser a chave para um novo preditor. Tióis têm atividade antioxidante e, durante processos oxidativos, podem ser transformados em moléculas diferentes, tais como o tiol-dissulfeto.^4^ Estudos já demonstraram que a razão tiol-dissulfeto é um marcador promissor de stress oxidativo.^5^^,^^6^

Erdoğan et al.^7^ estudaram a associação entre parâmetros de tiol de plasma e níveis de troponina em pacientes com síndrome coronária aguda (SCA) e a previsão de arritmia ventricular hospitalar.^7^

Foram estudados 191 pacientes diagnosticados com SCA, subdivididos em SCA sem supradesnivelamento do segmento ST (SCA-SSDST) e com supradesnivelamento do segmento ST (SCA-SDST). Além da dosagem de tiol nativo, dissulfeto, e a razão tiol-dissulfeto, outros parâmetros foram avaliados em um modelo univariado e no modelo de regressão logística, tais como LDL, idade, FEVE, potássio sérico, e níveis de magnésio, razão neutrófilo-linfócito (RNL), tempo de hospitalização, razão troponina-tiol nativo (RTTN) e troponina.

Não houve diferença nos níveis de tiol plasmático (nativo ou total), dissulfetos, razão tiol-dissulfeto nativo, razão tiol-dissulfeto total na comparação entre a SCA-SSDST e a SCA-SDST, demonstrando que ambos os diagnósticos são semelhantes em termos de grau de stress oxidativo. Da mesma forma, não se encontraram diferenças em relação à ocorrência de arritmias ventriculares.

Na análise da correlação de variáveis múltiplas com parâmetros de stress oxidativo, muitos demonstraram resultados significativos, mas com correlação fraca ou moderada. A melhor correlação foi observada com a dosagem de CK-MB massa com a razão de troponina-tiol nativo (r = 0,63; p<0,001) provavelmente devido à correlação desse biomarcador com a troponina.

Por último, foi realizada a regressão logística para prever arritmias ventriculares em três cenários: SCA-SSDST, SCA-SDST, e toda a população. Na SCA-SDST, o tiol nativo foi um preditor independente, enquanto a RTTN foi um preditor na SCA-SSDST. No caso de toda a população com SCA, ambos os marcadores (tiol nativo e RTTN) foram variáveis preditoras. Foi dada ênfase maior à RTTN, que apresentou uma boa área sob a curva (AUC = 0,783) com boa especificidade e sensibilidade para prever arritmias ventriculares.

Este é o primeiro estudo a demonstrar a correlação entre marcadores de stress oxidativo e arritmias ventriculares, destacando a RTTN. O principal marcador conhecido atualmente é a área de lesão do miocárdio, documentada pela fração de ejeção.^8^ Pacientes com fração de ejeção reduzida merecem atenção especial em relação à necessidade de desfibriladores implantáveis, devido ao risco de arritmias ventriculares.

Os níveis de troponina influenciam diretamente a RTTN e podem demonstrar lesão miocárdica aumentada e, portanto, maior risco de arritmias ventriculares. O presente estudo não apresenta a curva ROC para tiol nativo, embora ele também tenha sido considerado um marcador em regressão logística, limitando a avaliação desse marcador. Sabemos que a troponina é um marcador de prognóstico importante em pacientes com SCA^9^ e tem uma correlação com a área de dano miocárdico.

Por outro lado, este e outros estudos demonstraram uma correlação entre níveis de tiol e gravidade (escore GRACE), incluindo a ocorrência de eventos adversos.^10^ Além disso, Rajic et al.^11^ demonstraram que o nível de tiol sérico era um preditor independente de complicações relacionadas ao infarto, especialmente a disfunção ventricular.^11^ Portanto, surge a pergunta se o tiol é um preditor independente de arritmia ventricular ou outro marcador de lesão e disfunção ventriculares.

A dosagem de tiol sérico não faz parte da avaliação de pacientes com SCA. Portanto, são necessários estudos maiores para avaliar o impacto real da incorporação dessa tecnologia ao controle desses pacientes.
