# Supplementary material for: Níveis Altos de Tiol: Um Novo Marcador de Arritmias Ventriculares na Síndrome Coronária Aguda?
Source: Arq Bras Cardiol. 2021 Sep 1;117(3):474–5. [Article in Portuguese] doi: 10.36660/abc.20210602 (PMC8462949; doi:10.36660/abc.20210602)
Supplement: Translation

# Translation: Elevated Thiol Levels: A New Marker of Ventricular Arrhythmias in Acute Coronary Syndrome?

**Affiliations:** 1 Rio de Janeiro RJ Brazil Hospital Barra D'Or - Rede D'Or São Luiz, Rio de Janeiro, RJ - Brazil; 2 Rio de Janeiro RJ Brazil Universidade Federal do Rio de Janeiro - Instituto de Cardiologia Edson Saad, Rio de Janeiro, RJ - Brazil

**Keywords:** Sulfhydryl Compounds, Arrhythmias, Cardiac, Acute Coronary Syndrome

Ventricular arrhythmias in patients with acute coronary syndrome are not frequent, but they lead to a greater occurrence of complications and death.^1^^,^^2^ Its prevalence is higher in infarction with ST-segment elevation (STEMI) and has a worse prognosis when it occurs 48 hours after admission.^3^ Thus, a marker capable of predicting the occurrence of these events is welcome.

Three mechanisms justify the occurrence of arrhythmias in the acute phase of the infarction:

- Injured myocardium can be a substrate for the development of reentry circuits or a focus of increased automaticity;
- Triggering of arrhythmias secondary to heart rate variation;
- Modulating factors, such as electrolyte disturbances, autonomic dysfunction, persistent ischemia, or reduced left ventricular function.

Thus, the identification of markers that influence these mechanisms can be the key to a new predictor. Thiols have antioxidant activity and, during oxidative processes, they can be transformed into different molecules, such as thiol-disulfide^4^. Studies have already shown that the thiol-disulfide ratio is a promising marker of oxidative stress.^5^^,^^6^

Erdoğan et al. studied the association between plasma thiol parameters and troponin levels in patients with acute coronary syndrome (ACS) and the prediction of in-hospital ventricular arrhythmia.^7^

A total of 191 patients diagnosed with ACS were studied, subdivided into ACS without ST-segment elevation (SCA-SSDST) and with ST-segment elevation (SCA-SDST). In addition to the dosage of native thiol, disulfide, and the thiol-disulfide ratio, other parameters were evaluated, both in a univariate model and in the logistic regression model, such as LDL, age, LVEF, serum potassium, and magnesium levels, neutrophil-lymphocyte ratio (RNL), time to hospitalization, troponin/native thiol ratio (RTTN), and troponin.

There was no difference in plasma thiol levels (native or total), disulfide, native disulfide-thiol ratio, total disulfide-thiol ratio, and native thiol-total thiol ratio when comparing SCA-SSDST and SCA-SDST, demonstrating that both diagnoses are similar in terms of the degree of oxidative stress. Likewise, no difference was found regarding the occurrence of ventricular arrhythmias.

In the analysis of correlation of multiple variables with oxidative stress parameters, many showed significant results, but with weak or moderate correlation. The best correlation was observed with the CK-MB mass dosage with the troponin-native thiol ratio (r=0.63; p<0.001), probably due to the correlation of this biomarker with troponin.

Finally, logistic regression to predict ventricular arrhythmias was performed in three scenarios: SCA-SSDST, SCA-SDST, and the entire population. In SCA-SDST, native thiol was an independent predictor, while RTTN was a predictor in SCA-SSDST. In the entire ACS population, both markers (native thiol and RTTN) were the predictor variables. A greater emphasis was given to the RTTN, which presented a good area under the curve (AUC=0.783), with good specificity and sensitivity for predicting ventricular arrhythmias.

This is the first study to demonstrate the correlation of oxidative stress markers with ventricular arrhythmias, highlighting RTTN. The main marker currently known is the area of myocardial damage, documented through the ejection fraction.^8^ Patients with reduced ejection fraction deserve special attention regarding the need for implantable defibrillators, due to the risk of ventricular arrhythmias.

Troponin levels directly influence RTTN and may demonstrate greater myocardial damage and, therefore, greater risk of ventricular arrhythmias. The present study does not present the ROC curve for native thiol, although it was also considered a marker in logistic regression, limiting the evaluation of this marker. We know that troponin is an important prognostic marker in patients with SCA^9^ and has a correlation with the area of myocardial damage.

On the other hand, this and other studies have shown a correlation between thiol levels and severity (GRACE score), including the occurrence of adverse events.^10^ Furthermore, Rajic D et al. demonstrated that the serum thiol level was an independent predictor of infarction-related complications, especially ventricular dysfunction.^11^ Thus, the question arises whether thiol is an independent predictor of ventricular arrhythmia or another marker of ventricular damage and dysfunction.

Serum thiol dosage is not part of the routine evaluation of a patient with ACS. Therefore, broader studies are needed to assess the real impact of incorporating this technology in the management of these patients.
